# Promoting collaborative care of the human body after cessation of life: Ubuntu beyond death

**DOI:** 10.4102/curationis.v47i2.2629

**Published:** 2024-11-15

**Authors:** Simangele Shakwane, Dumisani B. Vilakati

**Affiliations:** 1 Department of Health Studies, College of Human Sciences, University of South Africa, Pretoria, South Africa; 2 Manzini Diocese, Catholic Church, Manzini, Eswatini

**Keywords:** body, death, ritualisation, culture, nursing care, Christianity, touch, Ubuntu

## Abstract

**Background:**

South Africa is a diverse country that promises equality, dignity, linguistic and cultural rights to all its citizens. Therefore, understanding the cultural, religious and nursing practices in caring for the deceased body is crucial to ensure meaningful integrated care of the deceased body and collective mourning and support within a community.

**Objectives:**

This study aimed to strengthen multidisciplinary collaboration and knowledge sharing on caring for the human body at all stages of life and beyond death using Ubuntu principles.

**Method:**

A qualitative exploratory-descriptive design was used to understand the meaning of the human body after cessation of life within African, Christian and nursing practice. The study was conducted in three countries in Southern Africa (eSwatini, South Africa and Zimbabwe). Snowball and purposive sampling techniques were used to recruit participants. In-depth telephonic and face-to-face interviews were conducted. The data were analysed thematically.

**Results:**

To provide comprehensive insights, themes from the three population groups were integrated. Four main themes emerged from the thematic analysis: (1) death as an end to physical life, (2) cleanliness of the deceased body, (3) ritualisation of death and (4) beyond death and burial.

**Conclusion:**

Strengthening multidisciplinary collaboration is vital to providing comprehensive care for the body and spirit of the deceased. Understanding cultural and religious rituals helps with collective mourning and support in the community.

**Contribution:**

Reflecting on the meaning of the deceased body and the respect given to it during the process of transition into the spiritual world through ritualisation.

## Introduction

The human body goes through various processes of life, birth, suffering and death. Caregivers are often confronted with the death and end of a patient’s life, and this experience is inevitable (Fridh 2014:307). The person is considered human in the context of a living human physical body and consciousness. Unfortunately, death is unavoidable for all living beings and human dignity at the end of life is undeniable (Bilgiç 2023:1388). Therefore, the termination of life occurs as a permanent cessation of brain function, characterised by the complete absence of consciousness and inability to breathe independently (Shemie et al. 2023:144). Biomedical death is the cessation of vital bodily functions, the continuation of neurological activity and the maintenance of a state of consciousness (Williams 2016:135). Death is a permanent and irreversible loss of consciousness caused by brain death. Brain death is an expression of the lack of blood and oxygen, leading to the brain cells’ deterioration and disintegration (Attoe 2023:316).

Death is a natural transition from the visible to the invisible or spiritual ontology, in which the spirit, the essence of the person, is not destroyed but moves to live in the realm of the spiritual ancestors (Baloyi & Makobe-Rabothata 2014). In hospital practice, if death is imminent, the patient is, if possible, moved to a single room in the private ward to protect the privacy of the patient as well as the family. If rooms are occupied, the patient’s death takes place behind closed curtains in the open ward (Hadders 2007:210). Death is a source of great sadness, despair and worry; therefore, patients want to die in dignity and peace, and be cared for according to ethical standards (Bilgiç 2023:1389).

Intimate care and touch do not end at the cessation of life. Nurses must maintain the dignity of the deceased patient’s body through the last office: identification, final washing and wrapping of the body. According to the South African Nursing Council (SANC 2022), section 3 (1) (n), nurses should ensure appropriate palliative and end of life in nursing care. The last office is the hospital’s final duty to the patient, with nurses officially exercising the final act of care and responsibility to the body (Williams 2016:135). The nurses should provide controlled care of the lifeless body. Through this process of the nurse’s final duty to the patient, the patient’s body is constantly exposed and touched. Touching is always seen as an act of reciprocity. Unfortunately, at this stage, the patient cannot consent to the final care of his or her body. The body can no longer be considered a private space; it is a realm of perpetual opening to other bodies and vulnerability (Zengin 2022:352). Therefore, it is of utmost importance that nurses treat the patient’s body with dignity and respect (Bilgiç 2023:1390) and acknowledge the condition the person was in life (Williams 2016:140).

Within South African legal premises, a person’s legal personality ends upon death, meaning that a deceased person has neither rights nor obligations (Heaton 2017). The right to bodily and psychological integrity is an important innovation in the Constitution of the Republic of South Africa of 1996 (Currie & De Waal 2018). The law protects the body of the deceased and regulates its disposal. This is done out of community interest, out of respect for the feelings and sensitivity of the deceased’s relatives and respect for the dead (Heaton 2017).

The religious view of human personality is linked to spirituality. According to religious belief, death is not the absolute end of existence, but rather a transformation into another as the souls of those who have died in Christ partake of their heavenly rewards (Sim 2015:147). This is related to the African metaphysical definition of death as transitional, that one survives bodily death (Attoe 2023:311). Death is an event that represents transcendence and transformation, a transition into eternal spiritual existence as an ancestor (Attoe 2023:317; Thomas 2021:7). Because the deceased person and their family are members of a community, death becomes a communal event in which the community supports the family in the grieving process.

### Ubuntu as a theoretical foundation

Drawing from Ubuntu characteristics of communal living, respect and dignity of a human person, death is seen as a community event. Ubuntu is an indigenous concept describing the affirming dignity of self and others in a shared humanity (Moyo 2021). This is summarised in the expression ‘umuntu ngumuntu ngabantu’ which means, a person is a person through other persons (Müller, Eliastam & Trahar 2019). South Africa is a diverse country with different religious, cultural and linguistic groups. Human transcendence and the transition into the spiritual world are a sacred time for the family and community to prepare for the necessary rituals. Therefore, the spirit of Ubuntu is often practised in African communities.

Ubuntu is the African idea of personhood and the self is defined and understood in relation to others (Baloyi & Makobe-Rabothata 2014). African beliefs are founded on the principles of collective interest of the group and that the person cannot exist alone. Therefore, incorporating traditional sources of community support is vital in dealing with death, life crises and events (De Beer & Brysiewicz 2017:22). Ubuntu is a collective ontology that emphasises intra-community relationships in which ancestors and future generations are part of a community; therefore, it is a continuous movement of the unfolding of the universe (Van Norren 2022:2791). It symbolises togetherness and caring for each other in a community; it upholds the need for secure social equilibrium, compassion, humanness and strong consideration of the other’s humanity (Kamga 2018:624). Thus, Ubuntu calls for a humanistic approach in which the needs and well-being of the community are considered more important and the principles of sharing, caring and compassion for others are at the forefront (Mangena 2016:64).

Southern African countries are diverse with multiple cultural and religious practices. This paper is part of a larger study that seeks to understand the African human body to advocate for holistic integrated care of the touched body. In this qualitative exploratory-descriptive phase, the authors sought to understand the importance of multidisciplinary approaches in caring for the human body of the deceased using the Ubuntu principles of dignity, respect and communal approach to death.

## Research methods and design

### Study design

For this phase of the study, a qualitative exploratory-descriptive design was used to understand the meaning of the human body after cessation of life within the African, Christian and nursing perspectives. The qualitative research design is a plan to collect and analyse evidence that will help the researcher answer the research question (Flick 2018). Exploratory-descriptive qualitative research is conducted to address a problem or issue that requires solution and understanding, thereby, providing insights into practical problems (Grove & Gray 2023).

### Setting

The study was conducted in three countries in Southern Africa (Eswatini, South Africa and Zimbabwe), specifically targeting religious and African Christian leaders. For nurse practitioners, the study was conducted on medical and surgical wards in two selected hospitals in Gauteng province.

### Study population and sampling

Three population groups participated in this study and are discussed respectively as follows:

Group 1: Religious Christian leaders – these were the priests and pastors of mainline churches such as the Catholic and Lutheran churches, including other Protestant churches and African Christian churches such as the Zionists. Participants were leaders in their respective churches, supported grieving families and conducted funeral rites for their church community. Their ages ranged from 37 to 59 years. Snowball sampling was used to recruit participants. In this sampling strategy, the second author identified the initial participants from the three countries (Eswatini, South Africa and Zimbabwe) and was then asked for names of other religious Christian leaders who shared the same relevant characteristics, leading to a chain of participants based on recommendation.

Group 2: African indigenous practitioners, the first author consulted with the first *gogo* who assisted with the recruitment of other participants who were African indigenous practitioners for more than 3 years and whose ancestry spirit permitted them to participate in the study. They were between 45 and 60 years old.

Group 3: Nurse practitioners – registered nurses employed in the two selected hospitals, with at least 3 years’ experience in medical and surgical units, and have provided the last office to the deceased human body. The nurse practitioners were purposively sampled. Purposive sampling is a technique for selecting individuals with specific knowledge or expertise about the topic under study (Grove & Gray 2023). The first author is an experienced registered nurse. After obtaining ethical clearance and approval from the two hospitals, she visited the eight medical and surgical units (four medical and four surgical), two from each hospital. The unit managers provided a 15 min morning slot after patient handover to explain the purpose, objectives and expectations for participating. The study information leaflet and the informed consent form were provided to the nurses interested in the study. They provided their cell phone numbers for further conversations and telephone interviews. The participants were 30–55 years old. In all, 16 nurse practitioners participated which was guided by data saturation.

### Data collection

In-depth interviews were conducted to understand the meaning and rituals of the deceased body from multiple perspectives. In-depth interviews involve inductive, open-ended questions, ranging from general to specific questions, in which participants provide long and detailed responses (Flick 2018). Data from the three different population groups were collected using interviews: face-to-face (*n* = 6), telephone (*n* = 16) and email (*n* = 8). All groups were asked three main questions, and probing was conducted to understand the information shared. Table 1 shows the questions asked of all groups.

**TABLE 1:** Interview guide for participants.

What significance does death have in your practice (nursing, Christian and African practice)? How is the human body prepared for the next phase of life after death? What rituals are performed to mark the final farewell to earthly life?

#### Telephonic interviews

Christian leaders – The interview guide was emailed to participants so that they could read and answer the questions. After the questions were answered, the participant emailed them to the second author, who reviewed the documents for completeness and meaning. The two authors read the answers independently for initial analysis. Participants whose responses required verification were contacted to clarify ambiguous information.

Nurse practitioners – Participants were recruited face-to-face in respective hospitals. The first author phoned each participant who provided a cell phone number to secure an appointment for the interview. The telephonic interviews were conducted during the evenings or days off. The participants signed the informed consent and emailed it to the researcher before the interview.

The interview lasted 15–20 min. The interviews were audio-recorded with the permission of the participants.

#### Face-to-face interviews

The first author consulted the first participant *(gogo),* who invoked the ancestors and requested their support in the conducting of the interviews. The study was explained in simple terms that were understandable to the participant’s ancestors. Three Nguni languages were used: siSwati, isiZulu and isiNdebele. After seven days, the first participant responded with an acceptable date and time for the interviews. During the seven days, she consulted with other African indigenous practitioners who were also interested in the study and permitted to participate. On the day of the interview, six African indigenous practitioners were present at the agreed venue. The interviews had to be completed on that particular day. The special room was used for the interviews; the altar was equipped with candles, incense, water and *ugwayi* (snuff) to ask the ancestors to bless the day and for discussions ‘ukunxusa amathonga’. The researcher explained the study and requested permission to ask questions and record the discussion, promising to ask questions with respect and present them honourably to their ancestors. All six African indigenous practitioners signed informed consent and data collection continued. Individual interviews were conducted with the elder *gogo*, who observed the interviews and listened to ancestral messages. Interviews lasted 20–45 min and were digitally recorded with participants’ consent.

### Data analysis

Thematic data analysis was used to identify, analyse and interpret patterns of meanings within the qualitative data (Clarke & Braun 2016:297). The authors used the four phases described by Vaismoradi et al. (2016) of thematic analysis. In the *initialisation phase*, the researchers read the verbatim transcripts and highlighted units meaning, coding was conducted and supported by the abstracts from the participants’ data. In the *construction phase*, the codes were defined, organised and compared for similarities and differences. This phase allowed the researchers to simplify the codes created so that they were translated into understandable concepts and experiences of the participants. In the *rectification phase*, the themes developed were described and compared to the literature to identify the existing knowledge. The *finalisation of themes phase*, supported the development of the storyline, in which the results were narrated by describing and linking various themes to the aim of the study.

### Trustworthiness

Trustworthiness refers to the degree of confidence in data, interpretation and methods used to ensure the quality of a study (Connelly 2016:435). Table 2 presents the trustworthiness principles and their application in the study.

**TABLE 2:** Trustworthiness principles and their application in the study.

Trustworthiness principles	Application
Credibility – Confidence in the truth of the study; it asks ‘How congruent are the findings with reality’ (Stahl & King 2020:27). A study is considered credible if the descriptions of human experiences are immediately recognised by individuals who share the same experiences (Cope 2014:90).	*Triangulation:* Three population groups were used to obtain multiple perspectives on the care of the deceased body. The findings were integrated to find comprehensive meanings and practices acceptable to the African context. The two researchers were experts in different fields (S.S. was a nurse practitioner knowledgeable about African indigenous practices and D.B.V. was a priest, scripture scholar and theologian).
	*Prolonged engagement:* This occurred during the recruitment of participants, where information about the study was shared with prospective participants. Telephonic conversations were also conducted to gain trust and establish an open relationship with participants. The interviews lasted 20–40 min and the researchers attempted to obtain as much information as possible through follow-up questions to ensure clarity and understanding.
	*Member check:* After data analysis, S.S. met with the African indigenous practitioners to confirm the accuracy of the information on relevant themes and sub-themes. Because participants’ identities were coded, it was not possible to identify the data per participant; this was done with a group and this also brought closure and a prayer of thanksgiving was done during this session.
Confirmability – is the neutrality or the degree findings are consistent and could be repeated (Connelly 2016:436). It refers to the researcher’s ability to demonstrate that the data represent the participants’ responses and not the researcher’s biases or viewpoints (Cope 2014:90).	An audit trail allows the readers to form a judgement about the quality and worth of the study (Hadi & Closs 2016:643). A detailed description of the research design, sampling, data collection and analysis were discussed. This allowed the authors to describe how conclusions and interpretations were established.
Transferability – is the extent to which findings are useful to persons in another setting, the reader can determine how applicable the findings are to their situation (Connelly 2016:435).	A detailed description of the context, location and participants studied was discussed. These included the context of the study (setting), inclusion and participants’ characteristics.
Authenticity – is the extent to which researchers fairly and completely show a range of different realities and realistically convey participants’ lives (Connelly 2016:435).	Appropriate and knowledgeable people were selected for the study with a rich, detailed description of the results to demonstrate the deep meaning of the phenomenon. Excerpts from participants were used to represent their voices.

Note: Please see the full reference list of this article: https://doi.org/10.4102/curationis.v47i2.2629.

### Ethical considerations

The study received an Ethical Clearance Certificate from the College of Human Sciences Research Ethics Committee (CREC) with reference number 90414357_CREC_CHS_202 from the University of South Africa. The principles of autonomy and informed consent were adhered to by informing participants about the nature of the study, its purpose, its benefits and how they will participate in the study. Before conducting the interviews, participants were requested to sign informed consent, and the nurse practitioners and Christian leaders emailed their informed consent forms. To protect their identity, all documents with personal information were saved in a password-protected folder. For the African indigenous practitioners, informed consent was collected by author 1 before the commencement of the face-to-face interviews. During telephonic interviews, MS Teams caller was used to record the interviews. Only voice recording was done; the participants were requested to consent to the recording. The participants were treated with respect and dignity. Their cultural and religious values were acknowledged and protected by not asking offensive questions, respecting time by conducting the interviews at the date and time agreed upon and reporting the findings accurately. All raw data were saved in the researchers’ computers in password-protected folders. Only the two researchers had access to the data. No identifiable information was disseminated, and each participant had a code.

## Results

Group 1: Church leaders: Eight (*n* = 8) Christian leaders participated in written and follow-up interviews (Table 4). The participants were from three countries: Three (*n* = 3) from South Africa, three (*n* = 3) from Eswatini (formerly known as Swaziland) and two (*n* = 2) from Zimbabwe. Four (*n* = 4) were priests and/or pastors from mainline churches (Catholic and Lutheran) and four (*n* = 4) were Africans from the Zionist churches. They were between 37 and 59 years old with church leadership experience of 5–25 years. They were fluent in English, siSwati and Shona.

**TABLE 3:** Integrated findings for the study participants.

Theme	Sub-theme
1. Death an end to physical life	1.1 Non-responsiveness to stimulation 1.2 Being called to eternal life 1.3 Transitioning to ancestorhood
2. Cleanliness of the deceased body	2.1 Maintaining the dignity and privacy of the body 2.2 Identification, washing and dressing up the body
3. Ritualisation of death	3.1 Announcement of death 3.2 Spiritual accompaniment 3.3 Fetching of the body and spirit home 3.4 Final farewell: Funeral rite
4. Beyond death and burial	4.1 Remembrance through identification and visiting the grave

**TABLE 4:** Group 1: Church leaders’ participant characteristics.

Participant’s code	Gender	Age (years)	Years of experience	Country	Church
ACL01-45-M	Male	45	20	Zimbabwe	ZCC
CL02-37-M	Male	37	7	Eswatini	Catholic
CL03-40-M	Male	40	10	South Africa	ZCC
ACL04-39-F	Female	39	10	Zimbabwe	ZCC
CL045-55-M	Male	48	16	South Africa	Lutheran
CL06-59-F	Female	59	20	Eswatini	ZCC
CL07-41-M	Male	41	8	Eswatini	Catholic
CL08-58-F	Female	58	15	South Africa	Pentecostal

Group 2: African indigenous practitioners: Six (*n* = 6) participants who practised African indigenous medicine participated in face-to-face in-depth interviews (Table 5). They were between 45 and 60 years old and were all females with more than 3 years in indigenous healing practice.

**TABLE 5:** Group 2: African indigenous practitioners’ participant characteristics.

Participant’s code	Gender	Age (years)	Years of experience
AFP01-50-F	Female	50	10
AFPL02-59-F	Female	59	15
AFP03-48-F	Female	48	8
AFP04-55-F	Female	55	17
AFP05-53-F	Female	53	12
AFP06-46-F	Female	46	10

Group 3: Nurse practitioners: Sixteen (*n* = 16) nurse practitioners participated in telephone in-depth interviews (Table 6). The majority of the participants were female (*n* = 13) and were between 30 and 55 years old. They had 3 years and more clinical experience in medical and surgical nursing care.

**TABLE 6:** Group 3: Nurse practitioners’ participant characteristics.

Participant’s code	Gender	Age (years)	Years of experience	Unit or ward
PN01-35-F-MU	Female	35	5	Medical
PN02-30-F-SU	Female	30	3	Surgical
PN03-47-F-SU	Female	47	5	Surgical
PN04-40-M-MU	Male	40	6	Medical
PN05-36-F-MU	Female	36	5	Medical
PN06-30-M-SU	Male	30	3	Surgical
PN07-30-F-MU	Female	30	3	Medical
PN08-32-F-MU	Female	32	4	Medical
PN09-34-F-SU	Female	34	5	Surgical
PN10-50-F-MU	Female	50	20	Medical
PN11-52-F-SU	Female	52	22	Surgical
PN12-35-M-SU	Male	35	3	Surgical
PN13-49-F-MU	Female	49	7	Medical
PN14-55-F-MU	Female	55	23	Medical
PN15-49-F-MU	Female	49	6	Medical
PN16-50-F-MU	Female	50	15	Medical

### Integrated findings of the study

The findings from the three groups were integrated to present comprehensive meanings and interpretations. Table 3 presents the themes and sub-themes:

#### Theme 1: Death an end to physical life

Death ends physical existence; in nursing, care responsibility ends once a person no longer responds to physical stimuli and the layout is completed. The family and community take on the task of carrying out the prescribed rituals. In the African and Christian belief systems, death does not end a person’s existence. Christianity describes death as a call to eternity with God and African cultures view death as a transition to ancestral role. Three sub-themes describe death according to the three population groups.

**Sub-theme 1.1: Non-responsiveness to stimulation:** Nurses are guided by the medical definition of death when the patient does not respond to neurological (pupils do not respond to light), cardiac (no heartbeat) and respiratory (no breathing) stimuli. The participants described the death as follows:

‘We [*nurses*] consider the patient dead when he [*or*] she stops breathing and the vital signs monitoring machine stops working either by making an alarming noise or all patient information becomes flat and moves to zero. We inform the attending doctor who confirms or certifies the patient dead.’ (PN01-35-F-MU)

‘Death is confirmed by the doctor who does the final certification patient’s death. But most times the senior nurse on duty is informed to double-check the observations such as the heartbeat, respiration and pupil constriction …’ (PN07-30-F-MU)

Nurse practitioners rely on patients’ physical responses, such as breathing and eye pupil dilation. They are assisted by technology in determining death by checking and monitoring vital signs.

**Sub-theme 1.2: Being called to eternal life:** Christian leaders point to death as a call to eternal life, a call back to God the Creator. Physical death ends earthly life and paves the way to spiritual life. The participants described the death as follows:

‘As Christians regardless of domination, we believe that death is a pathway to eternal life. As one is from God when they die, they return to God their creator …’ (CL03-40-M)

‘… because God created us, so when our time is over from this earthly life, we go back to God. The body and spirit separate, the spirit goes back to God.’ (CL02-37-M)

When a person dies, the body and spirit separate and return to their Creator. The soul is a spiritual life personified, it does not die but returns to God while the body lies buried on earth.

**Sub-theme 1.3: Transitioning to ancestorhood:** The meaning of death is a controversial discourse in African culture as the cessation of physical life does not mean the end of a person’s existence as the preservation of the human body is important. The person exits the earthly life to join the realm of the ancestors. The participants described the death as follows:

‘In African culture, a person does not die, but transcends to the realm of ancestors. It is a return home to the bigger family and join them in spirit.’ (AFP01-50-F)

‘An African person does not die: [*isilwane siyafa*] – an animal dies, but [*umuntu uyashona*] meaning going beneath the earth to connect with other ancestors.’ (AFP03-48-F)

The idea is that a person does not die, but transcends to a spiritual life of ancestors who watch over and protect their families on earth.

#### Theme 2: Cleanliness of the deceased body

Physical cleanliness confers a state of dignity and integrity on the deceased. Even if the person cannot respond, washing and dressing the deceased body ensures privacy and proper preservation of identity. The nurses, family members and delegated community members are responsible for preparing the body for the final farewell to eternal life. These activities are discussed by participants in the following sub-themes.

**Sub-theme 2.1: Maintaining the dignity and privacy of the body:** Nursing practitioners discuss their duties and responsibilities in preserving the dignity of the deceased patient through privacy. The patient’s final journey may occur in an open ward with other patients and, if possible, transferred to a private ward. No matter what environment the patient is in, closing the curtain or doors serves to ensure their physical privacy:

‘When the patient dies, we close the curtain and close the door in a private room so that other patients will not see the deceased person.’ (PN07-30-F-MU)

‘When the patient becomes very critical, like gasping [*difficult breathing*]. If there is space in the sideward so that the patient and family have privacy during the difficult time. We screen for privacy to allow the patient to die peacefully.’ (PN10-50-F-MU)

Once the patient dies and is still warm, the body is prepared for eternal sleep by being placed in the supine position (lying flat on the back with all limbs straight). The eyes are also closed:

‘Immediately after death, we remove excess linen such as pillows and extra blankets. The patient is placed in a supine position, where they are straight and facing up. The neck is supported with a small pillow to keep it straight. The wet cotton will is used to close the eyes while the body is still warm.’ (PN13-49-F-MU)

A nurse’s duty to care for a patient’s physical condition immediately after death cannot be overemphasised. Preparing the body for eternal sleep can allow families to retain fond memories of their loved one’s final moments and peaceful closure to the earthly life.

**Sub-theme 2.2 Identification, washing and dressing up the body:** Washing the body is critical in maintaining the patient’s dignity. Cleanliness of the body is a sign of readiness for the next life and respect for the deceased person. Before the final preparation, the body is identified by the nurse practitioners and delegated family members.

Correct identity is crucial when a patient is unable to identify himself or herself. The nurses use name tags to write the patient’s details:

‘We use multiple name tags for the patient during layout [*procedure to prepare a patient for transfer to mortuary*]. On the tag, we write the name and surname of the patient, hospital number and ward where the patient was admitted with bed number. Name tags are placed on the forehead, chest, arms and toes. This is done to ensure that the right patient is received by the right family. This assists when facial identification cannot be done.’ (PN03-47-F-MS)

After the announcement of death, the family delegates go to the hospital mortuary to identify their loved one. The elders who know the physical characteristics of the person are the ideal ones as they will be able to do the correct identification:

‘For us Africans, to bury the correct person is important as it will be a disaster to send a wrong person to our ancestors. The close elders of the family go to the hospital to see if the deceased person is their family member or not. If it is theirs, the family responsible person will sign the papers so that they can register the death in home affairs, and get a death certificate to prepare for the funeral.’ (AIP05-53-F)

The African Christian leaders emphasised the importance of gender care, in which a similar gender attends to the body of the deceased church member:

‘The deceased person is dressed by members of the church in the church uniform. A male member is dressed by males as they know how to dress him properly and a female is dressed by females as well.’ (ACL02-59-F)

During the bathing and dressing of the deceased body, respect is maintained as he or she is wrapped in a white garment:

‘The body is bathed and dressed in a white garment. Respect is observed. In some cases the old woman within the church is the one who manages it [*body of the deceased*].’ (ACL-39-F)

‘The corpse of the dead body is treated with the utmost respect. The deceased body is bathed by church leaders and dressed in white garments. The body is wrapped in a white cloth and buried without a coffin.’ (ACL01-45-M)

The body is washed for cleansing of all physical dirt and dressed in white symbolising purity. The body is prepared for its final rest.

#### Theme 3: Ritualisation of death

A series of prescribed rituals are conducted to prepare the deceased for final rest and the family in their mourning process. This theme discusses the family and community journey from the announcement of death, spiritual accompaniment and support, fetching the body and spirit home and final farewell through funeral rites.

**Sub-theme 3.1: Announcement of death:** Sharing the news of a family’s death serves to promote collective grief and support within the community. Death is a communal event because the deceased person is a member of a community; therefore, community support is important:

‘When a member of the community dies the parish community will celebrate a mass for the dead member and also burry the member using the Christian burial rite.’ (CL02-37-M)

‘When a person dies, the death is immediately reported to the parish priest so that funeral arrangements can be made. The extended family and community are also informed about the death so that they can help in the arrangements.’ (CL04-55-M)

Within a community, men help dig the grave. This is an important task because these men begin preparing the grave site early in the morning, thus relieving the burden on the family. Therefore, announcing death to the community becomes a form of information sharing and preparation for this task:

‘In rural communities, we still depend on community men to dig the grave. When “isifo” [*death*] has been announced to the neighbours and community leaders, the community men leader visits the family to enquire about funeral arrangements so that he can gather men who can assist in digging the grave.’ (AIP05-53-F)

**Sub-theme 3.2: Spiritual accompaniment:** The Christian leaders emphasised the importance of spiritual support during the mourning process. Spiritual accompaniment is provided through prayers, guidance and counselling for the family members:

‘Spiritual accompaniment of the close relatives is done to support them as they prepare for the final interment of the deceased.’ (CL03-40-M)

‘The Church should assist the African community to restore the communal care of each member of the community, especially during their loss. Spiritual support through prayer and counselling is important to ease the pain of losing the loved one.’ (CL02-37-M)

Losing a loved one is a painful experience; therefore, the relatives need to be encouraged and strengthened through prayers. As family members prepare to say their final farewell to their loved ones, they need to be strengthened spiritually so that they can send off their loved one in peace.

**Sub-theme 3.3: Fetching of the body and spirit home:** If a person dies away from home, the body and spirit should be connected before the funeral. The elders go to the place where the person died to fetch the spirit using ‘Umlahlankosi’ a branch of a special indigenous tree where the spirit of the deceased attaches itself to the thorns on the branches once called and asked to come home. The spirit of the deceased attaches itself to the thorns of the branches:

‘In most South African cultures, the body and spirit must be one during burial. On the day when the body is fetched from the mortuary, the spirit is also fetched from the place where it is separated using Umlahlankosi. The elders will call upon the spirit of the deceased and ask it to come home with them. If this ritual is not done the spirit of the person will roam around endlessly with no chance of connecting with their ancestors.’ (ACL02-59-F)

‘Sometimes relatives of the deceased patients come to the ward to fetch the spirit of their loved one with a branch. We allow them to do it outside the ward to respect the patients who are in the ward and the one occupying the bed.’ (PN13-49-F-MU)

It is important to understand the cultural belief systems of patients and families to support them in their grieving process and accompany their loved ones according to the prescribed rituals. At the same time, nurses must protect the patients in the ward by not exposing them to this ritual.

**Sub-theme 3.4: Final farewell: Funeral rite:** Once all family rituals are completed, the community joins the family in a final farewell. The funeral rites prescribed by various Christian communities are used. The elements of prayer and blessing are used to usher the deceased person to God. Water and incense are used to bless the body for its final journey:

‘… the body is taken to church for Requiem Mass, the coffin is sprinkled with holy water as a remembrance of their baptism and the incense is also used.’ (CL04-55-M)

‘The body of the deceased person is usually buried into the ground in a place selected by the family and relatives … The deceased is accompanied by the priest, family, friends and community where he or she lived. The coffin is taken to the graveyard, where all the family rituals take place where he [*or*] she is buried with the tomb being blessed at the end.’ (CL02-37-M)

The rites for the celebration of funerals foresee several rituals that should be done on the body. Upon arrival at the church, the coffin and the body of the deceased are sprinkled with holy water to remember the deceased person’s baptism (The Rites of the Catholic Church 1976). Arriving at the gravesite, the priest does a farewell prayer which speaks, not of throwing away the body, but of committing it to the earth. Once the body is placed in the grave, the priest followed by the close relatives picks up some soil with their bare hands and throws it into the grave indicating that the deceased is in harmony with the earth even in death.

#### Theme 4: Beyond death and burial

This theme speaks of life after death and affirms life beyond the grave. How family members keep alive and respect the memory of their loved ones is of utmost relevance. The sub-theme focusses on the grave, identifying the grave and visiting to remember those gone before us.

**Sub-theme 4.1: Remembrance through identification and visiting the grave:** The grave is marked with the deceased person’s name(s) and surname of the deceased person, as well as the date of birth and death as a form of identity so that the family can know where their loved one is sleeping:

‘… close relatives are the one’s who visit the tomb for cleaning and talking with their loved ones. Some bring flowers and their life problems, even prayer.’ (AFP01-50-F)

‘The relatives visit the graveyard and clean it as a sign of respect for the dead … because through death the spirit lives.’ (CL04-55-M)

A cemetery is considered a sacred place that must be protected because when they encounter problems, they talk to their ancestors about solutions. Therefore, the grave is treated with respect and dignity.

## Discussion

The purpose of this article was to understand the importance of multidisciplinary approaches in caring for the human body of the deceased using Ubuntu principles of dignity, respect and a communal approach to death. Four main themes emerged from the data collected from nurse practitioners, Christian leaders and African indigenous practitioners, which were: (1) Death an end to physical life, (2) cleanliness of the deceased body, (3) ritualisation of death, and (4) beyond death and burial.

### Death was seen as an end to physical life

Nurse practitioners defined death as a lack of response to physical stimulation. This definition is consistent with biological death, which refers to the irreversible process of cellular and tissue functions leading to cardiopulmonary failure caused by the predominant cessation of spontaneous breathing, heartbeat and circulation (Le Roux-Kemp 2013:77). According to the *South African National Health Act*, the moment of death is defined as brain death (South Africa 2003). The *Birth and Death Registration Act 51 of 1992* requires that a medical practitioner must issue a notification of death stating the cause of death for the Home Affair to register a death and provide a death certificate to the family. The Christian and African views of death suggest that there is spiritual life either with God or ancestors. For Christians, when a person dies, he or she is called to eternal life, which is life in heaven with God the Creator (Hamilton et al. 2017:665). Death is the end of the human lifespan, which essentially represents the separation between soul and body (Hartin 2022:279). In African indigenous practice, death was described as transitioning to an ancestral role. The African traditions view death as an act of reunification with ancestors; it is seen as a transition from one mode of existence to another, changing from a physical person to a spiritualised life (Mosima 2023:159). It is going home where one belongs, rejoining the old relatives for eternal life (Zungu 2021). Death is not the end, but merely a transition from the physical world to the world of spirits and ancestors (Potocnik & Adum-Kyeremen 2022:89).

The loss of physical life liberates the individual and allows them to access the spiritual world as a disembodied spirit (Attoe 2023:319). They are in a state of collective immortality in the company of the spirit (Baloyi & Makobe-Rabothata 2014).

### Cleanliness of the deceased body

The cleanliness of the body is crucial, regardless of whether it is alive or deceased. The participants attached importance to maintaining physical dignity during the final care of the deceased body. Rituals such as washing, dressing and being looked at by relatives are crucial for the final farewell to the deceased. The washing ritual is an intimate component of care as it involves close physical contact, exposure of the body and the transgression of some established boundaries of the social body (Vilakati & Shakwane 2024:2). The washing of the body allows families to identify, gaze and touch their loved ones. It allows them to embody and express their grief through tactile care work (Zengin 2022:360). Shakwane (2023) found that patients who were hospitalised during the coronavirus disease 2019 (COVID-19) pandemic were not afraid to die but feared to die dirty, emphasising the importance of cleanliness of the body before crossover or transitioning to ancestorhood. The body needs to be cleansed to return to its Creator God or ancestors as pure as at birth (Lobar, Youngblut & Drooten 2006:47). Zengin (2022) emphasises that washing the deceased with careful care and respect is a symbol of respect for the dead body as a newborn baby for another universe accessible in the afterlife.

Nurse practitioners who participated in the study described their responsibilities during the last office as cleaning the patient, positioning them correctly and final identification. Shortly after death, the patient is positioned in an acknowledged social form, eyes and mouth closed. Washing the body occurs within the first few hours after death, while the dead body may still be warm and more akin to a living body; soiled, blood stains and other signs of suffering from the dead body are removed (Hadders 2007). While nurses wash the deceased body, they wash the patient’s private parts with a towel, protecting the privacy and integrity of the deceased (Hadders 2007:220). During the last office, the patient’s body is both dead and naked; identity has been removed and recreated as a corpse by the addition of shrouds and labels (Quested & Rudge 2003:589). Therefore, the deceased body should be treated with respect and dignity.

Family members or designated community members are responsible for cleanliness at the time of death. They pay their last respects to the deceased and prepare the body for the transition to the next life.

### Ritualisation of death

During the mourning period, religion and culture play an important role as they determine the process to be followed. Traditional sources of community support are critical in dealing with death and crisis. Ritual is a specific behaviour or activity that gives symbolic expression to certain feelings and thoughts of the actor or actors individually or as a group (Makgahlela et al. 2019:95). They are beneficial towards the process of acceptance and recovery (Woods 2014). After death, the deceased goes through ceremonies to remember the deceased and to assist the soul in going to heaven (Lobar et al. 2006:48). Rituals are also representations of cultural performances and rites of passage which mark people’s life experiences (Baloyi & Makobe-Rabothata 2014).

The nurse practitioners are responsible for notifying relatives about the death of their loved one and discharging the care of patient belongings (Quested & Rudge 2003:579). Because death is a community event, the family announces to the extended family and community for collective grief. In African communities, death is a social event that initiates a person into a social afterlife (Makgahlela et al. 2019:99), thus advocating for collective support and mourning.

Body and spirit should be connected at the funeral. Participants in this study emphasised the importance of fetching the spirit ritual performed when the person dies away from their home. The ritual of fetching the spirit refers to a traditional act that aims to retrieve the spirit or soul of a deceased person from the place of death and bring it home where it can rest in peace (Jiyane, Phiri & Peu 2012). This is done to prevent deceased wandering spirits (Khosa-Nkatini, Wepener & Meyer 2020:2). In Zulu culture ‘Umlahlankosi’, known as the Buffalo Thorn tree, is used to summon the spirit of the deceased (Hans 2015). The delegated family members use the branch where the person died and speak to the deceased to come home. The spirit of the deceased is believed to ride on the branch from the place of death to the burial site (Cann 2018). In this ritual, the dedicated person carries the branch and does not speak throughout the journey.

The burial rituals are carried out so that cohesion, peace and solidarity can be restored within the family and society (Zungu 2021:4). Current practice in Christian churches shows a similar respect and reverence for a person’s body after death. According to the Code of Canon Law of the Roman Catholic Church:

Ecclesiastical funerals, by which the Church seeks spiritual support for the deceased, honours their bodies, and at the same time brings the solace of hope to the living, must be celebrated according to the norm of the liturgical laws. (Code of Canon Law 1983)

It is important to emphasise that a Christian funeral is not just the burial of a person’s body in the ground but an act of honouring the body and bringing solace and hope to the living.

### Beyond death and burial

The loss of physical life frees the individual and allows them access to the spiritual world as a disembodied spirit (Attoe 2023:319). They are in a state of collective immortality in the company of the spirit (Baloyi & Makobe-Rabothata 2014). It is believed that the burial of the loved one has a connection to their resting place and ensures the continuity of life beyond the grave. An individual life in a spirit world, receiving a new body that acts as an ancestor, is only possible for individuals who have lived a meaningful life (Ekore & Lanre-Abass 2016). Visiting the grave allows one to reflect on the life they shared with their loved ones come to terms with the new life without the person, and ask for success and wellbeing (Ngubane 2004).

African and Christian spirituality supports the connection between the living and spiritual worlds. The belief that death leads one to God or ancestors gives the family hope as they continue to remember their loved ones by visiting the cemetery. In isiZulu, a burial is considered ‘*ukungcwaba*’ or ‘*ukufihla*’ – Africans do not separate themselves from their dead but rather exist in relation to the world of their ancestors (Maphela 2021:6).

Nurses deal with death and dying experiences on a daily basis and provide physical care with respect and dignity. Based on the insights presented, each practitioner has a specific role to play in the person’s transition to the next life. Knowledge sharing is crucial to understanding patient and family cultures and practices for integrated collaborative care of the body. Every practice has accepted values and norms that are adhered to. Therefore, creating a safe space for the sharing of knowledge and experiences is critical to identifying common practices and divergences that need to be reconciled for deceased persons and families.

### Strengths and limitations

The study was limited to three Nguni cultures: Zulu, Swati and Ndebele. Because this is a qualitative exploratory-descriptive study, the results cannot be generalised. To generalise the results, different cultural groups and research designs should be used in different contexts.

### Implications and recommendations

The study results recommend strengthening collaborative practice in multidisciplinary practice. This collaboration aims to improve the quality of cohesion in the care of people from the physical end of life to the transition to spiritual life. Nurse practitioners should understand the patient’s cultural and religious practices to provide quality care. This can only happen when knowledge is shared openly as well as their common reasons and disagreements. The body of a deceased patient should be treated with respect and dignity during the last office procedure. African indigenous practice becomes important, especially in fetching the spirit. Diverse cultural practices should be respected and appreciated.

## Conclusion

This article presented the importance of caring for the naked human body after cessation of life, that is, the practice of Ubuntu beyond death. Collaboration between various stakeholders, including the nurse practitioners, family members and religious and community leaders is key in caring for the deceased as each person or group plays their role. The Christian and African view death not so much as a cessation of life but its continuation. For Christians, death is a transition to God and the African worldview is a transition to the realm of the ancestors. This, therefore, requires that the body be treated with dignity and kept clean. Leaders in African churches consider this so important that they even wash the body in the mortuary, while mainline Christian churches sprinkle holy water on the body during funeral ceremonies as a sign of cleansing. Respect and dignity of the body therefore go beyond the termination of physical life. The burial of the person also bears witness to this, as the rituals speak of ongoing communication between the deceased and those still living. The burial site becomes not just only a place for the dead, but also a place where constant communication can take place between the living and the deceased. Respect and dignity for the naked and lifeless human body is therefore the best way to practice Ubuntu towards the dead, and this also applies to life even beyond the grave.
